# Comorbid Medical Issues in X-Linked Ichthyosis

**DOI:** 10.1016/j.xjidi.2022.100109

**Published:** 2022-02-17

**Authors:** Lucija Brcic, Georgina H. Wren, Jack F.G. Underwood, George Kirov, William Davies

**Affiliations:** 1 School of Psychology, Cardiff University, Cardiff, United Kingdom; 2 MRC Centre for Neuropsychiatric Genetics and Genomics, School of Medicine, Cardiff University, Cardiff, United Kingdom; 3 Division of Psychological Medicine and Clinical Neurosciences, School of Medicine, Cardiff University, Cardiff, United Kingdom; 4 Neuroscience and Mental Health Research Institute, Cardiff University, Cardiff, United Kingdom

**Keywords:** Mb, megabase, STS, steroid sulfatase, XLI, X-linked ichthyosis

To the Editor

X-linked ichthyosis (XLI) is a rare dermatological condition characterized by abnormal desquamation and retention hyperkeratosis (Fernandes et al., 2010). It is caused by deficiency of the enzyme steroid sulfatase (STS), most frequently arising from genetic deletions on the X chromosome (Fernandes et al., 2010). XLI overwhelmingly affects males, although female carriers of XLI-associated variants may exhibit dry skin (Fernandes et al., 2010).

In males, XLI has been associated with an increased likelihood of cryptorchidism, innocuous corneal opacities, and neurodevelopmental/mood disorders and associated traits (Chatterjee et al., 2016; Fernandes et al., 2010; Rodrigo-Nicolás et al., 2018). Female carriers can exhibit delayed or prolonged labor during childbirth as a consequence of reduced placental STS activity and subsequent lower estrogen production and can exhibit a similar, although milder, constellation of neurodevelopmental/mood traits to affected males (Cavenagh et al., 2019). By using the large UK Biobank resource of approximately 0.5 million middle-aged individuals drawn from the UK general population and examining hospital-assigned International Classification of Diseases, Tenth Revision diagnoses, we recently identified atrial fibrillation/flutter as being diagnosed more frequently in males carrying deletions of 0.8–2.5 megabase (Mb) around *STS* than in male noncarriers (Brcic et al., 2020). To call copy number variants, anonymized genotype data were downloaded as raw CEL files from the UK Biobank website (https://www.ukbiobank.ac.uk/) and analyzed using Affymetrix Power Tools and PennCNV-Affy software (Wang et al., 2007), taking into account participants’ genotypic sex. Identifying comorbid medical conditions in XLI is key to ensuring optimal multidisciplinary care for affected individuals and for providing better genetic counseling.

Our original UK Biobank analysis screened for International Classification of Diseases, Tenth Revision diagnostic codes present in >2.5% of male deletion carriers and in >2,500 males in the overall sample; theoretically, this analysis might have missed conditions that are common in deletion carriers (i.e., present in at least 1 in 40 individuals) but are comparatively rare in the population as a whole. In this study, we report two additional diagnostic codes that are present in >2.5% of male XLI carriers but are generally rare (i.e., present in <2,500 of UK Biobank males). We have been prompted to present these findings now after recent discussions with males with XLI and female carriers in online patient support groups in which subchorionic hematoma during pregnancy and a high frequency of nosebleeds have been described by multiple deletion carriers.

“Haemorrhage and haematoma complicating a procedure, not elsewhere classified” was reported significantly more frequently in male deletion carriers than in male noncarriers (3.5% vs. 0.5%, *P* = 0.009) and more frequently in a mixed-sex sample of deletion carriers than in a mixed-sex sample of noncarriers (1.8% vs. 0.5%, *P* = 0.007) (Table 1). Seven deletion carriers that were assigned this diagnostic code had a mean deletion size of 1.57 ± 0.10 Mb, with a 0.97 Mb consensus deletion interval spanning the *HDHD1*/*PUDP*, *MIR4767*, and *STS* genes (X:6,740,007-7,713,010, GRCh37 genome build). “Palmar fascial fibromatosis [Dupuytren]-Hand” was reported significantly more frequently in male deletion carriers than in male noncarriers (3.5% vs. 0.6%, *P* = 0.018) (Table 1). The four deletion carriers that were assigned this diagnostic code had a mean deletion size of 1.48 ± 0.20 Mb, with a 0.89 Mb consensus deletion interval spanning the *VCX3A*, *HDHD1*/*PUDP*, *MIR4767*, and *STS* genes (X:6,489,969-7,384,404, GRCh37 genome build). We did not identify any deletion carriers within our sample who had received a diagnosis of both “Haemorrhage and haematoma complicating a procedure, not elsewhere classified” and “Palmar fascial fibromatosis [Dupuytren]-Hand.” No individuals presenting with the aforementioned medical conditions had received a hospital International Classification of Diseases, Tenth Revision diagnosis of ichthyosis or had self-reported a previous diagnosis of blistering/desquamating skin disorder during an interview at the UK Biobank Assessment Centre; however, it should be noted that <5% of all male deletion carriers in UK Biobank self-reported such a condition, indicating substantial underdiagnosis or misdiagnosis of XLI in this cohort (Brcic et al., 2020).

**Table 1:** ICD-10 Descriptive Codes Identified in >2.5% of Male XLI Deletion Carriers and <2,500 Males Overall in UK Biobank Sample

ICD-10 Descriptive Code	UK Biobank Code	Group	Number Affected (%)	Fisher’s Exact Test Result
Palmar fascial fibromatosis [Dupuytren]-Hand	85340	Male deletion carriers	3/86 (3.5)	*P* = 0.018
		Male noncarriers	1,214/190,577 (0.6)	
		Female deletion carriers	1/312 (0.3)	*P* = 0.364
		Female noncarriers	329/227,862 (0.1)	
		Deletion carriers (male and female combined)	4/398 (1.0)	*P* = 0.062
		Noncarriers (male and female combined)	1,543/418,439 (0.4)	
Haemorrhage and haematoma complicating a procedure, not elsewhere classified	142880	Male deletion carriers	3/86 (3.5)	*P* = 0.009
		Male noncarriers	946/190,577 (0.5)	
		Female deletion carriers	4/312 (1.3)	*P* = 0.109
		Female noncarriers	1,315/227,862 (0.6)	
		Deletion carriers (male and female combined)	7/398 (1.8)	*P* = 0.007
		Noncarriers (male and female combined)	2,261/418,439 (0.5)	

Abbreviations: ICD-10, International Classification of Diseases, Tenth Revision; XLI, X-linked ichthyosis.

To date, there is little published evidence for a biological link between STS deficiency and bleeding problems. However, such a link is plausible: in adult tissues, *STS* is most highly-expressed in the arterial vasculature (GTEx Portal, 2021); the STS substrate, cholesterol sulfate, acts as an endogenous modulator of hemostasis (Sanchez et al., 2021); and circulating levels of a second STS substrate, dehydroepiandrosterone sulfate, have been associated with thrombus formation under certain conditions (Harrington et al., 2017; Roetker et al., 2018). Moreover, antepartum hemorrhage has been reported in a female carrier (Attenburrow et al., 1984). It is also possible that the apparent increased risk of hematoma/hemorrhage in deletion carriers is simply a correlate of these individuals being exposed to more surgical procedures, or more invasive surgical procedures than noncarriers, for example, orchidopexy or emergency cesarean section.

Dupuytren contracture is a progressive fibrosing disorder arising from nodules and thickened cords in the palmar fascia that may lead to contractures of the fingers (Stewart and Nascimento, 2021). There is existing evidence that individuals with other forms of ichthyosis may exhibit contractures, thickening of the palmar fascia, and constricting bands of skin (Craiglow, 2013; Palmer and Louis, 1976), and acute inhibition of STS in mice has been associated with increased expression of the fibrosis-related genes *Ccn2* (*Ctgf*) and *Ccn3* (*Nov*) (Humby et al., 2016; Rebolledo et al., 2021).

The associations described in this study may be clinically relevant but are clearly provisional, with no accounting for multiple testing; hence, they will require replication and further investigation, for example, examining the extent to which they overlap with dermatological phenotypes. If confirmed, they will shed light on the biological basis of hemostasis and fibrotic conditions. We suggest that clinicians consider the possibility of the aforementioned (and related) conditions when evaluating males with XLI and female carriers.

### Data availability statement

Datasets related to this article are available on application to UK Biobank (https://www.ukbiobank.ac.uk/).

## ORCIDs

Lucija Brcic: http://orcid.org/0000-0002-8115-3644

Georgina H. Wren: http://orcid.org/0000-0001-9179-136X

Jack F.G. Underwood: http://orcid.org/0000-0003-1731-6039

George Kirov: http://orcid.org/0000-0002-3427-3950

William Davies: http://orcid.org/0000-0002-7714-2440

## Author Contributions

Conceptualization: WD; Data Curation: LB, JFGU, GK, WD; Formal Analysis: LB, JFGU, GK, WD; Funding Acquisition: WD; Investigation: LB, JFGU, WD; Methodology: LB, JFGU, GK, WD; Project Administration: WD; Supervision: WD; Writing - Original Draft Preparation: WD; Writing - Review and Editing: LB, GHW, JFGU, GK

## Conflict of Interest

The authors state no conflict of interest.
